# Predictors and Moderators of Long-Term Outcome of Persons at Clinical High Risk for Psychosis: Methods and Preliminary Data

**DOI:** 10.1093/schbul/sbaf133

**Published:** 2025-08-26

**Authors:** Kristin S Cadenhead, Leda Kennedy, Heline Mirzakhanian, Jean Addington, Carrie E Bearden, Tyrone D Cannon, Ricardo E Carrión, Matcheri Keshavan, Daniel H Mathalon, Diana O Perkins, William Stone, Elaine F Walker, Scott W Woods

**Affiliations:** Department of Psychiatry, UCSD, San Diego, CA 92093-0810, United States; Department of Psychiatry, UCSD, San Diego, CA 92093-0810, United States; Department of Psychiatry, UCSD, San Diego, CA 92093-0810, United States; Department of Psychiatry, Hotchkiss Brain Institute, University of Calgary, Calgary, AB T2N 4N1, Canada; Departments of Psychiatry and Biobehavioral Sciences and Psychology, Semel Institute for Neuroscience and Human Behavior, UCLA, Los Angeles, CA 90095, United States; Department of Psychology, Yale University, New Haven, CT 06511, United States; Department of Psychiatry, Donald and Barbara Zucker School of Medicine at Hofstra/Northwell, Hempstead, NY 11549, United States; Department of Psychiatry, Harvard Medical School at Beth Israel Deaconess Medical Center and Massachusetts Mental Health Center, Boston, MA 02215-5400, United States; Department of Psychiatry, UCSF, San Francisco, CA 94143, United States; SFVA Medical Center, San Francisco, CA 94121, United States; Department of Psychiatry, University of North Carolina, Chapel Hill, NC 27514, United States; Department of Psychiatry, Harvard Medical School at Beth Israel Deaconess Medical Center and Massachusetts Mental Health Center, Boston, MA 02215-5400, United States; Departments of Psychology and Psychiatry, Emory University, Atlanta, GA 30033, United States; Department of Psychology, Yale University, New Haven, CT 06511, United States

**Keywords:** Prodrome, schizophrenia, affective psychosis, death, incarceration

## Abstract

**Background and Hypothesis:**

Despite significant advances in our understanding of the clinical high risk (CHR) for psychosis state, the longer-term outcomes (5+ years) and the trajectory of diagnoses, symptoms, and psychosocial function have been seldom investigated.

**Objective:**

Here we describe the methods for “Predictors and Moderators of Long-Term Outcome of Persons at Clinical High Risk for Psychosis,” an ongoing study that is being conducted across North American Prodrome Longitudinal Studies sites that included *n* = 2184 past participants (1999-2018).

**Study Design:**

The aims are to: (1) perform long-term assessments of individuals who previously met CHR criteria, (2) determine the 5+ year psychotic conversion rate and use previously collected longitudinal clinical, functional, neurocognitive, and biomarker data to predict longer term outcomes, and (3) investigate predictors of long-term clinical/functional outcome in CHR participants who did not convert to psychosis.

**Study Results:**

Preliminary results from the first *n* = 504 participants demonstrate that 60% of those who previously met CHR criteria are still symptomatic. Eighteen percent of past participants converted to psychosis, half in the original studies and the remainder since last evaluated. Of those who converted to psychosis, the majority met criteria for an affective psychosis, consistent with the high rate of affective disorders (70%) in the non-converted group. An additional 7% of past participants died, substantially higher than the general population.

**Conclusions:**

These early data highlight the potential of how this dataset, when combined with baseline data, can be used to answer new questions about the life course of high-risk youth and how we might intervene early to improve their long-term outcome.

## Introduction

Psychotic illnesses are neurodevelopmental disorders with evidence of pathological changes in utero; premorbid neuromotor and neurocognitive abnormalities; subsyndromal psychotic symptoms in the prodromal period (clinical high risk, CHR); and full manifestation of a psychotic syndrome during late adolescence or early adulthood. CHR research over the past 2+ decades has provided (1) important insights into risk factors for conversion to full psychotic illness within a 2-3 year period, (2) the development of a “Psychosis Risk Calculator,”^1^ (3) biomarkers linked to psychosis risk,^2–5^ and (4) evidence of dynamic brain changes^6^ that are likely present before the onset of illness and continue to evolve into first episode psychosis and more chronic forms of psychosis. Despite these advances in our understanding of the CHR state, the longer-term outcomes (5+ years), including diagnoses, symptoms, and psychosocial function have been seldom investigated in the CHR population.^7^^,^^8^ Longer-term follow-up of CHR individuals provides a unique and rare opportunity to investigate the full trajectory of illness from CHR → First Episode → Chronic Illness.

The CHR criteria identify a heterogeneous population with not only sub-syndromal psychotic symptoms but neurocognitive deficits, comorbid mood, anxiety, and trauma related symptoms, along with significant social and role functioning problems.^9^ Meta-analyses show that 20%-30%^10^ develop psychosis within 2 years and 1/3 of known psychotic conversions occur after 2 years.^11^ The question of how many conversions occur after 5 years has not been extensively studied in a prospective longer-term follow-up design. Retrospective studies suggest that the prodromal phase of illness can last up to 20 years,^12^ but it is unclear which early CHR characteristics predict a later vs early psychotic conversion, affective vs non-affective psychosis, or good vs poor outcome.

The majority of individuals who meet CHR criteria do not develop overt psychosis within 2 years but demonstrate outcomes ranging from complete remission to continued symptoms and functional impairment.^13^ We applied group-based multi-trajectory modeling on 2 year longitudinal CHR data and identified 3 early trajectory groups based on symptoms and functioning over time (improvement, moderate impairment, severe impairment) that corresponded to good, fair, or poor 2 year outcomes, respectively.^14^ It is not known whether the outcomes remain stable at 5+ years and if the early poor trajectory predicts a later psychotic conversion.

Evidence exists for multiple biomarker abnormalities in CHR.^2^^,^^4^^,^^5^^,^^15–17^ CHR youth show deficits in neurocognition,^15^ regional cortical gray matter,^16^ event related potentials (ERP),^5^^,^^17^ as well as higher polygenic risk scores,^18^ inflammatory markers^2^ and cortisol,^4^ relative to comparison subjects. Biomarkers also predict psychotic conversion^4^^,^^5^^,^^16^^,^^17^ and functional outcomes^5^^,^^16^^,^^19^^,^^20^ at 2 years. It is not known whether biomarkers predict 5+ year conversion and longer-term outcomes with greater predictive power.

Given the maturity of the CHR research field across multiple academic centers that are part of the North American Prodromal Longitudinal Studies (NAPLS) Consortium, we have identified thousands of participants meeting rigorous diagnostic criteria with known short-term outcome and rich biomarker data. As part of a study entitled “Predictors and Moderators of Long-Term Outcome of Persons at Clinical High Risk (CHR) for Psychosis” (also known as Long-Term Follow-Up [LTF]), we have a unique opportunity to follow up both individuals who converted to psychosis and those who did not in order to investigate disease prediction, progression, and moderating factors.

The specific aims are to: (1) Perform long-term (5-20 year) diagnostic, symptom, and functional assessments of up to 2000 individuals who previously met CHR criteria. (2) Determine the 5+ year psychotic conversion rate and use previously collected clinical, functional, neurocognitive, and biomarker data, collected at multiple time points over 2-4 years to predict longer-term diagnostic and functional outcomes of individuals who convert to psychosis. (3) Investigate predictors of long-term clinical/functional outcome in CHR participants who did not convert to psychosis and test whether outcomes are influenced by treatment and substance use.

**Figure 1:**
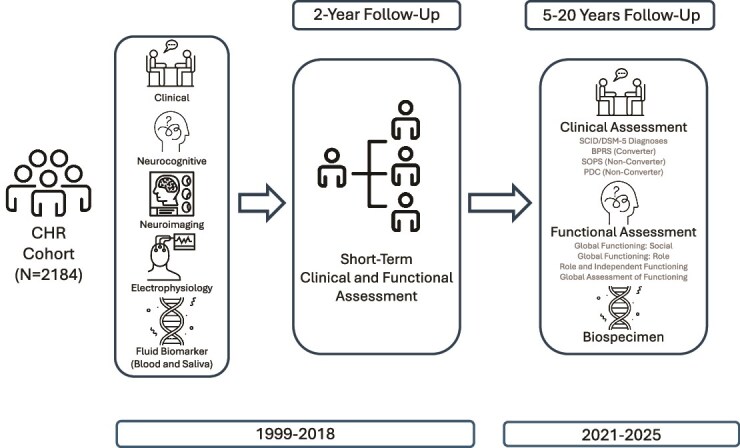
Study Design.

Here, we describe the methods of the LTF study (Figure 1) and characterize the preliminary sample of participants for what was originally slated to be a 4-year study between 2021 and 2025. Given study delays, primarily related to the COVID-19 pandemic, we are extending recruitment through August 2025.

## Methods

Nine sites across North America participated in the initial NAPLS consortium: the University of California San Diego, University of California Los Angeles, Emory University, Harvard University at Beth Israel Deaconess Medical Center, North Shore-Long Island Jewish Zucker Hillside Hospital, University of North Carolina Chapel Hill, University of Calgary, Yale University, and University of California, San Francisco. The NAPLS 1-3 studies,^21–23^ OMEGA3 trial,^24^ ReGroup study,^25^ and individual site studies have been conducted over the course of 2 decades across the various sites. Each study followed a longitudinal follow-up structure with demographic, clinical, functional, neurocognitive, and biomarker data collected at baseline and 2-6 month intervals for a total of 24-48 months. Individuals meeting CHR criteria participated between 5 and 22 years prior to the LTF study and comprised a clinically and demographically diverse sample consistent with the known heterogeneity of CHR populations. Here, we outline the general methodology of the original longitudinal studies and then our LTF methods to recontact and interview past participants to assess their current and interim status since their last research follow-up.

### Initial Recruitment and Sample Populations

Participants recruited into the CHR studies across the NAPLS sites were identified and recruited from surrounding communities through a variety of strategies and pathways. These individuals were generally help-seeking, and either self-referred for care and/or were referred by health, community, or school-based providers for research participation.^21–23^

### Initial Sample and Inclusion/Exclusion Criteria at Baseline

All individuals recruited to participate in the initial studies met the original inclusion criteria: (1) male or female, age 12-30 years; (2) understood and signed an informed consent (or assent for minors) document in English; (3) met criteria for CHR per the Criteria of Psychosis-Risk Syndrome from the Structured Interview for Psychosis-Risk Syndromes (SIPS).^26^ And did *not* meet any of the following: (1) current or lifetime Axis 1 psychotic disorder, including affective psychoses; (2) impaired intellectual functioning (IQ < 70); however those with an IQ in the 65-69 range were included if Wide Range Achievement Test reading >75; (3) past or current history of a clinically significant central nervous system disorder that may contribute to CHR symptoms or confounded their assessment; (4) substance dependence in the past 6 months; and (5) the diagnostic prodromal symptoms are clearly caused by a Diagnostic and Statistical Manual (DSM disorder, including substance use disorders. Other non-psychotic DSM disorders were not exclusionary (eg, substance abuse disorder, major depression, anxiety disorders, personality disorders), as long as the disorder does not account for the diagnosis of prodromal symptoms.

A total of 2184 participants were involved in NAPLS 1-3, OMEGA3, ReGroup, and other studies across all sites (Supplementary Table SS1). Of those, 1279 individuals agreed to be recontacted for future studies when originally consented (Supplementary Table SS2).

Baseline biomarker data including electrophysiological, neurocognitive, neuroimaging, inflammatory, and genetic data were collected as part of the NAPLS 1-3 and other studies across participating sites (Supplementary Table SS3).

### LTF Study Recruitment and Population

Centralized Institutional Review Board (IRB) approval was obtained to find, recontact, and interview past CHR participants using a variety of methods. Referencing guidelines for LTF in clinical research,^27^ the recruitment methodology of the LTF study involved contacting former participants, some of whom provided consent at the time of their initial participation in research to be contacted for future studies. In most of the initial studies, participants had the opportunity to indicate preference to be recontacted on their informed consent form. Recontacting methodology followed a pipeline format and utilized a variety of strategies to establish contact with eligible participants. Research staff, when establishing contact lists, identified past participants who explicitly indicated that they did not wish to be recontacted and were not approached. Attempts were made to contact all other past participants.

For those who did not indicate whether they wished to be recontacted (in some cases this was due to participant oversight when filling out the informed consent, eg, the participant left this question blank), research staff send a letter with information about the study via post with the institutional letterhead and a return stamped envelope. The letter includes the name of at least 2 staff members who worked with that participant at the time of their initial participation in research. If this attempt by mail is unsuccessful, research staff begin calling the last known phone number of the participant and any individual listed as a person to contact in the participants original research file. Once contact is established, research staff explain the proposed study and remind the individual/contact of their past participation and what will be involved.

For the participants who explicitly agreed to be recontacted at the time of their initial participation, research staff first attempt to reach them through the phone numbers and email addresses that they had initially provided. If participants listed a secondary contact or caregiver’s contact information, research staff attempt to make contact with this individual. If these initial methods are not successful, research staff send posted letters to the participant and listed contacts. Where appropriate, research staff used more official sources such as hospital records, Department of Motor Vehicles (DMV), social security administration, marriage, and divorce records to obtain contact information for participants. Social media and other internet searches are a final step in the contact pipeline. If unable to find contact information via previously mentioned methods, research staff send direct messages via social media platforms such as LinkedIn and Facebook *only* if at least 2 identifiers (eg, photo of the participant, name, hometown, date of birth) can be confirmed. Paid internet search programs such as Spokeo and Been Verified can be utilized to search participants by name if all other methods of recontacting are unsuccessful. Recruitment efforts such as fliers, advertisements, and public service announcements were made to the relevant communities, so participants who remain in the same communities are aware of the proposed study. Other sources of information regarding past participants were also explored including death and incarceration records.

### LTF Clinical and Functional Assessment

LTF interviews are primarily conducted via secure teleconferencing given the ongoing COVID pandemic at the time the study started. Our existing database includes basic demographics (age, sex, ethnicity, immigration status, parental SES, and education), family history of mental illness and history of adversity from the Childhood Trauma Questionnaire^28^ collected as part of NAPLS 1-3 and other site studies. Interim information is collected on education, marital status, employment, financial status, living arrangements, brain injury, psychiatric contacts and hospitalizations, aggressive history or police contact, medical conditions, medication, and other treatment history. The Structured Clinical Interview for DSM5 (SCID) is used to confirm or diagnose new disorders; the SIPS^26^ to determine if the participant still meets criteria for CHR or the criteria for conversion to psychosis are met. The Scale of Attenuated Psychotic Symptoms (SOPS) from the SIPS is also used to measure current prodromal symptom severity^26^ and the Brief Psychiatric Rating Scale (BPRS)^29^ is used to assess psychotic symptom severity in those individuals who converted to psychosis. Depression is assessed with the Calgary Depression Scale,^30^ and substance use with the Alcohol and Drug Use Scale^31^ as well as the Daily Sessions, Frequency, Age of Onset, and Quantity of Cannabis Use Inventory. To determine the impact, time and length of occurrence and severity of new stressful environmental events we again use the Peri Life Events Scale^32^ and violence risk is assessed with the HCR-20 (Historical Clinical Risk Management).^33^ Functional achievement is determined by Global Functioning (GF:Social and GF:Role) scales^34^ and Global Assessment of Functioning (GAF).^35^ We established reliability and monitoring procedures for the diagnostic, symptom, and functional evaluations^22^ used in the collection of original data and follow-up evaluations.

For the individuals who converted to psychosis, we had clinical consensus meetings to determine the DSM5 psychotic diagnosis (based on SCID, medical records, and informant history) and whether it was an Affective (Bipolar, Major Depressive, or Schizoaffective Disorder) vs Non-Affective (Schizophrenia, Schizophreniform, Delusional Disorder) Psychosis. Duration of Untreated Psychosis (DUP) was defined as the time in weeks from psychosis onset to the start of the first adequate treatment of psychosis. The onset of psychosis corresponded to the criteria for “POPS” (Presence of Psychotic Symptoms criteria) on the SIPS^26^ to differentiate between the attenuated positive symptoms that are part of the CHR criteria and full psychosis. The date of first treatment for psychosis was defined as date of initiation and adherence to an adequate dose of antipsychotic medication. Remission Criteria^36^ was defined as a score of ≤3 (on a scale ranging from 1 to 7) simultaneously on 7 BPRS items (Grandiosity, Suspiciousness, Hallucinations, Unusual Thought Content, Conceptual Disorganization, Blunted Affect, Mannerisms/Posturing) for at least 6 months. Recovery Criteria^37^ was defined as meeting the symptomatic remission criteria plus functional criteria on the GAF (>70), Global Functioning Role and Social Scales (>6) in the normal range for 2 years.

For those individuals who previously met CHR criteria and did not convert to psychosis, symptom severity and duration cut points were used to define 3 outcome groups based on criteria developed by Addington et al.^38^: (1) Remission—scores of 2 or less on all 5 positive symptoms on the SOPS scale; (2) Symptomatic but not currently meeting criteria CHR (symptoms have not increased in the last year); and (3) Prodromal progression—currently meet criteria for CHR on the SIPS. Psychosis-Risk Diagnostic Criteria (Brief Intermittent Psychotic Symptom Prodromal State, Attenuated Positive Symptom Prodromal State or Genetic Risk and Deterioration Prodromal State) including current status specifiers (Current, Previous, Current Persistence, Partial Remission, Full Remission)^39^ was determined for each non-converted past participant.

### Treatment History

Extensive history of resource utilization is assessed in the present LTF study and will be compared with participants’ reported resource utilization at the time of their initial participation. Because treatment could alter the association between predictors of outcome and the natural course of symptoms and functioning, research staff carefully document treatment received since participants’ last research assessment using the Resource Utilization Log that was developed for NAPLS 1-3 studies. This log involves identifying and recording each medication course since the participant’s last visit as defined by specific medication-dose combination, as well as the specific start and stop date. For each psychosocial treatment that the participant reports, study staff code the type of treatment, start and stop dates, and the number of sessions of treatment received.

### Biospecimen Collection and Storage

For the estimated 25% of individuals who did not provide a blood sample at the time of initial participation, a blood sample is collected and sent to the NIMH repository as done during the previous studies. A maximum of 50 mL of blood will be collected at the visit. DNA isolated from peripheral blood cells will be analyzed with the Illumina Global Screening Array Multi-Disease at the Broad Institute. Analysis of raw data follows the RICOPILI pipeline. Further quality control includes determination of cryptic relatedness with KING. When available population-relevant genome-wide association studies for polygenic risk score construction (eg, NeuroGAP-Psychosis) will be utilized.^40^ Blood samples will be securely stored for future studies. Chain of custody is documented by use of bar-coded and ID labeled blood collection tubes and bar-coded storage tubes, recorded in the central database for this study.

### Statistical Analyses

When data collection for the LTF study is complete and combined with all baseline data of the *n* = 2184 past participants from previous studies, we plan to investigate any selection bias in recruitment to determine generalizability of results. The number of individuals we were able to find and agreed to be interviewed will be compared to those who we did not find or refused interview on a variety of demographic and clinical variables. We are specifically interested in whether there was a difference between the number we found who had converted to psychosis in the parent studies vs those who did not. In addition, it is possible that there were differences across diagnostic groups, sites, ethnicity, sex, and other clinical variables.

Planned analyses to address the specific aims and hypotheses for the LTF will involve investigation of trajectories for conversion to psychosis that will be modeled using generalized linear mixed-effects models (GLMM) as well as weighted generalized estimating equations (WGEE). Predictors in these models will include the subject risk level based on the psychosis-risk calculator, polygenic risk scores for schizophrenia and bipolar disorder, biomarkers and corresponding timepoints, and relevant interactions controlling for all other covariates. Changes in strength of prediction over time will be assessed using linear contrasts. Importantly, the widely used psychosis-risk calculator developed within the NAPLS studies will be validated for long-term follow up using similar methods to those used in the original validation and replication studies being sure to use the same variables and weights to calculate the predictive power of the calculator.^1^ To verify the validity of the psychosis-risk calculator, the predictive power of the long-term vs short-term ratings will be compared through the Harrel’s C index, or the area under the receiving operating characteristic curve.

To evaluate whether the early persistent poor functioning trajectory identified while CHR will predict 5+ year conversion, trajectories will be developed using 2 different methods based on the original study data. First, Latent Class Growth Mixture Modeling (LCGMM) will be employed to determine different trajectories of functioning based on symptom measures and functioning over earlier periods of the study (eg, 2 year). Next, symptom severity and duration will be evaluated to define 3 trajectories based on original data for the non-converted CHR syndrome group.^14^^,^^38^ Different early trajectories to predict later psychotic conversion using GLMM/WGEE with a logit link will be used, where psychotic conversion (a binary indicator) will be the dependent variable, and group and time, (along with their appropriate interactions) will be the main predictors controlling for all other covariates. Original trajectories will be used to create group membership and then model conversion for each group and show that rates of conversion differ significantly in longer term follow-up. GLMM/WGEE will be employed with a generalized logit link to predict affective vs non-affective psychosis at outcome using baseline predictors, where affective vs non-affective psychosis will be the outcome, and DUP, gender, and polygenetic risk score will be the predictors, controlling for covariates. If a baseline variable predicts both affective and non-affective psychoses, directionality, and strengths of prediction will be assessed using appropriate linear contrasts. GLMM/WGEE with a logit link will be utilized to predict remission vs more chronic course of illness as outcome, and baseline symptoms and biomarkers, along with their appropriate interactions, will be the predictors, controlling for covariates.

Furthermore, LCGMM will be used as a first step as described above to predict clinical/functional outcomes. Outcomes will be treated as a 3-level categorical response and will use baseline symptoms, environmental, and biomarker data as predictors using GLMM/WGEE with a generalized logit link, where premorbid functioning, neurocognition, brain age gap, target P3b, and double-deviant MMN ERP amplitudes and their 2-way interactions with treatment and substance use moderator variables will serve as the predictors. Strengths of prediction for each predictor and moderation effects by treatment and substance use will be tested using appropriate linear contrasts and interpreted, along with directions of association, in support of the hypothesis.

For the current methods, paper we present descriptive data of the sample collected as of March 2025. We plan to extend recruitment during a no cost extension period through August 31, 2025.

## Results

### Demographics and Historical Information

The LTF data collected and entered as of March 2025 include *n* = 504 past CHR participants who were interviewed, *n* = 84 (16.7%) of whom converted to psychosis and *n* = 420 (83.3%) who remained nonpsychotic, whose demographics and historical information are outlined in Table 1. The converted sample was older, had a higher rate of unemployment, was more likely to have a family history of psychosis, more likely to be on an antipsychotic, to be receiving psychosocial intervention, to have a history of psychiatric hospitalization, a history of violence and cannabis misuse (*P*’s < .05-.001). The non-converted sample was younger, more likely to be on psychotropics other than antipsychotics and to have a history of non-violent offenses (*P*’s < .05-.001).

**Table 1:** Long Term Follow-Up Sample Characteristics

	**Non-converted LTF** **(*n* = 420)**	**Converted LTF (*n* = 84)**	χ^2^/t	*P*
Follow-up period years (SD)	9.2 (3.5)	10.3 (3.5)	1.6	NS
Age LTF years (SD)	28.3 (6.3)	30.1 (6.6)	2.5	.013
Sex (%F)	51%	41%	2.9	NS
Ethnicity (%Hispanic)	19%	18%	0.1	NS
Race (%Caucasian)	57%	57%	8.5	NS
Single/never married (%)	72%	77%	3.0	NS
Unemployed >1 year (%)	13%	29%	15.7	.003
Living independently (%)	57.2%	52.2%	11.8	.068
Education years (SD)	14.9 (2.5)	14.5 (2.6)	1.2	NS
Family history of psychosis	7.1%	13.0%	26.9	.046
Current antipsychotic (%)	10.5%	22.6%	60.0	<.001
Current other psychotropic (%)	52.1%	33.3%	60.0	<.001
Current psychosocial Tx(%)	35.2%	56.6%	58.3	.003
Psychiatric hospitalization (%)	10.8%	30.0%	14.0	<.001
History of trauma (%)	82.1%	78.6%	5.4	NS
History of violence (%)	15.7%	18.1%	17.1	.002
History non-violent offense (%)	23.0%	18.1%	16.8	.002
Current tobacco use	23.8%	30.0%	2.9	NS
Ever used cannabis	72.3%	78.1%	1.1	NS
Current cannabis use (%)	36.7%	37.4%	10.8	.013
Current cannabis misuse (%)	7.0%	14.5%		
**Scale of psychosis risk symptoms mean (range)**				
Total positive symptoms	6.62 (0-22)			
Unusual thought content/delusional ideas	1.86 (0-5)			
Suspiciousness/persecutory ideas	1.67 (0-5)			
grandiose ideas	0.44 (0-5)			
Perceptual abnormalities/hallucinations	1.51 (0-5)			
Disorganized communication	1.14 (0-5)			
**Total negative symptoms**	**6.79 (0-25)**			
Social anhedonia	1.49 (0-6)			
Avolition	1.55 (0-6)			
Decreased expression of emotion	0.67 (0-6)			
Decreased experience of emotions and self	1.13 (0-6)			
Decreased ideational richness	0.42 (0-6)			
Occupational functioning	1.54 (0-6)			
**Brief psychiatric rating scale mean (range)**				
BPRS total score		47.04 (24-97)		
Somatic concerns		2.44 (1-7)		
Anxiety		3.51 (1-7)		
Depression		2.82 (1-7)		
Suicidality		1.58 (1-5)		
Guilt		2.25 (1-7)		
Hostility		1.89 (1-7)		
Elevated mood		1.68 (1-6)		
Grandiosity		2.17 (1-7)		
Suspiciousness		2.95 (1-6)		
Hallucinations		2.75 (1-7)		
Unusual thought content		3.17 (1-7)		
Bizarre behavior		1.50 (1-5)		
Self-neglect		1.70 (1-5)		
Disorientation		1.24 (1-4)		
Conceptual disorganization		2.00 (1-5)		
Blunted affect		1.91 (1-5)		
Emotional withdrawal		1.63 (1-5)		
Motor retardation		1.20 (1-3)		
Tension		1.65 (1-4)		
Uncooperativeness		1.16 (1-4)		
Excitement		1.46 (1-5)		
Distractibility		1.75 (1-5)		
Motor hyperactivity		1.49 (1-7)		
Mannerisms and posturing		1.18 (1-4)		
**Current global functioning mean (SD)**				
GAF	66.80 (14.40)	57.08 (15.03)	5.57	<.001
Role	6.93 (2.37)	6.07 (2.60)	2.96	<.001
Social	7.25 (1.43)	6.63 (1.66)	3.53	<.001

In addition to the past CHR individuals who consented to interviews, we have found information through public records or a family member about an additional *n* = 62 individuals who participated in past CHR studies (bringing the total with information to *n* = 566). Of those, *n* = 40 (7% of total sample) have died, 5 of which are known suicides, 3 had converted to psychosis and 1 was incarcerated. A total of *n* = 14 (2.5% of total sample) were incarcerated and an additional *n* = 6 developed a psychotic episode (conversion rate of total sample 15.9%). Three were in the military suggesting they have better functioning and are likely non-psychotic.

### Current Clinical State at LTF of Past CHR Participants

**Figure 2:**
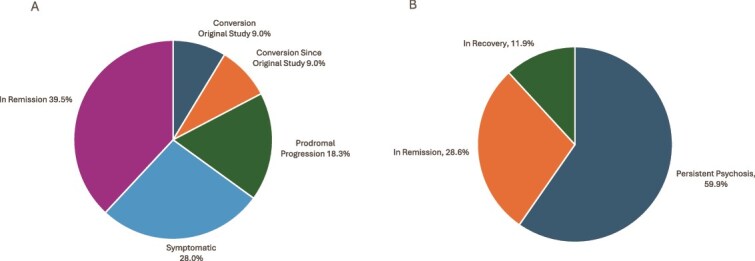
(A) Current Clinical State at Long-term Follow-up of All Past CHR Participants. (B) Long-term Outcome of CHR Participants Who Converted to Psychosis.

Of those *n* = 510 past participants with known clinical state data (including the *n* = 6 additional participants known to have converted to psychosis), *n* = 45 (11.2%) converted to psychosis during the original studies while an additional *n* = 46 (11.0%) were found to have converted to psychosis either during the original study, but had been lost to follow-up, or after 5 years from the original study baseline date (Figure 2A). The mean time to conversion of *n* = 82 who provided this information was 1008.6 (range 16-7000) days and DUP was 95.9 (range 0-2273) days. Of those *n* = 84 past participants who we know converted to psychosis and were fully assessed, *n* = 50 (59.5%) were actively psychotic at the time of the LTF interview, *n* = 24 (28.6%) had symptomatic remission and *n* = 10 (11.9%) were in recovery (Figure 2B).

There were *n* = 414 past CHR participants who did not convert to psychosis (with current clinical state data) during the follow-up period. Of those, *n* = 215 (51.9%) still met symptomatic criteria while *n* = 199 (48.1%) were symptomatically in remission. Of those who still had attenuated psychotic symptoms, *n* = 141 (34.1%) had persistent symptoms but no longer met CHR criteria (no recent increase in symptoms) and *n* = 74 (17.9%) still met CHR criteria with an increase in symptoms in the last year (Figure 2A).

### Symptoms and Functioning

Symptom and functioning ratings for both those who converted to psychosis and those who did not are shown in Table 1. There was a full range of symptoms present across the SOPS and BPRS on clinical assessment; the converted sample had lower global, role, and social functioning compared to the non-converted sample (*P*’s < .001).

### DSM Diagnoses

**Figure 3:**
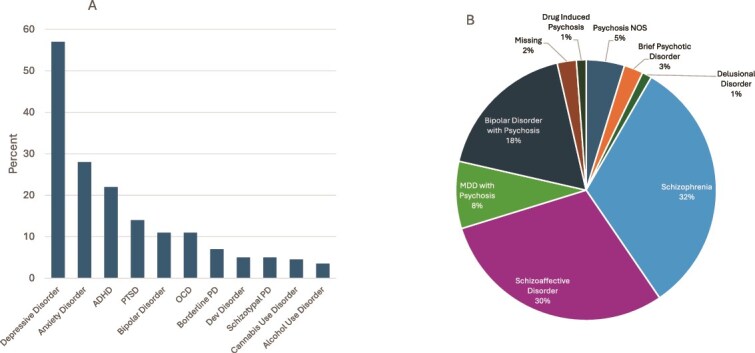
(A) Co-morbid DSM5 Disorders in Non-converted CHR Participants at Long-term Follow-up (LTF). (B) DSM5 Psychotic Diagnoses of Converted CHR Participants at LTF.

The DSM5 diagnoses of all past CHR participants are shown in Figure 3. This includes the DSM5 diagnoses of those who did not convert to psychosis (Figure 3A) as well as the final psychotic diagnoses of those who converted to psychosis (Figure 3B). Altogether, over 80% of the non-converted sample had a DSM diagnosis of a mood, anxiety, trauma, or attention disorder. Among the 84 past participants who converted to psychosis, the majority (56.0%) met criteria for an affective psychosis diagnosis, primarily schizoaffective disorder (29.8%) or bipolar disorder with psychotic features (17.9%), while less than half met criteria for a non-affective psychotic disorder (33.3%), primarily schizophrenia (32.1%).

## Discussion

To our knowledge, this is among the first long term follow-up studies of CHR youth.^7^^,^^8^ The NAPLS consortium remains one of the largest research initiatives to date focused on early identification and characterization of youth at CHR for psychosis. Throughout the NAPLS consortium’s several iterations over the past 2 decades as well as associated studies at the NAPLS sites, researchers have identified clinical, functional, neurocognitive, and biomarker profiles that elucidate risk and provide insights into the neurobiological basis of psychosis in young people who meet the CHR criteria.

Our preliminary LTF data demonstrates that 60% of youth who previously met CHR criteria are still symptomatic or have converted to psychosis at 5-20 year follow-up while 40% are in symptomatic remission. Slightly more than half are living independently and 25% are married. Eighteen percent of past participants had converted to psychosis at LTF. Half of those who converted did so in the original 2-3 years studies and half were newly identified converters at LTF. Given that the conversion rate in NAPLS 1 was 35%,^41^ 16% in NAPLS 2^1^ and 15% in NAPLS 3,^42^ it is likely we found fewer of the past participants we already knew converted (9% of the interviewed sample) compared to those who did not convert in the original studies. Importantly, an additional 9% of the sample we identified as now having a psychotic diagnosis were not previously known to have converted to psychosis. Given that we have found information on approximately 25% of the sample so far, we estimate that we have similarly found less than 25% of past and new psychotic conversions. When we complete data collection and combine the LTF data with baseline data from the parent studies it will be possible to determine whether there was an ascertainment bias in finding more past non-converters versus converters.

The majority of those individuals who converted to psychosis remained actively psychotic at LTF and over 55% met criteria for an affective psychosis vs a non-affective psychosis such as schizophrenia. This is a much higher rate of affective psychosis than reported in previous meta-analyses.^43^ The non-converted sample also had a high rate of comorbid mood disorders at LTF, along with anxiety disorders, suggesting that the comorbid conditions may account for the continued attenuated symptoms of psychosis in this population. It is of course possible that the individuals we were able to find, and who agreed to be interviewed, were more likely to have an affective disorder but the picture that continues to emerge in the CHR literature is that our current criteria identifies individuals who are indeed at high risk of serious mental illness but not specifically schizophrenia spectrum illness. Therefore, although it is tempting to model CHR and others with “psychotic-like experiences” as on a continuum with schizophrenia, a better conceptualization is to perhaps view this population as pluripotent and at risk for mental health problems in general.^44^ This finding of a high prevalence of affective disorders is consistent with observations of the potential therapeutic value of antidepressants in CHR adolescents.^45^ Given that treatment, prognosis, and rate of recovery can vary according to whether there is an affective component, the ability to predict course of illness and future diagnosis, as we have the potential to do in LTF, is crucial. Future analyses will investigate predictors of not only conversion but affective and non-affective illness. It will also be possible to investigate whether comorbid mood disorders contribute to poor outcomes and whether they mediate or confound psychosis risk. Future studies will need to consider how intervention strategies may differ depending on likely CHR outcome.

A surprising number (7%) of past CHR participants were found to have died since participation in past studies compared to the transitional age youth death rate in the general population globally of 1%^46^ and <0.08% in the United States.^47^ Although a small number of deaths are known to be by suicide, accident, or medical cause, the majority are unknown. Given that suicide, homicide, and accidents are among the leading causes of death in transitional age youth,^48^ it is possible that the prevalent comorbid conditions including affective disorders and substance misuse contributed to the early deaths. Although we may never know the cause of death of the majority in this sample, we have recently reported that in the NAPLS 3 sample 30% report a history of suicidal ideation, plan, or attempts.^49^ The high rate of violence, non-violent offenses, and incarceration are also somewhat surprising. Only 2 of the 9 sites had information on incarcerations in their sample, since state laws vary, so it is possible this number (*n* = 14, 2.5%) is even higher given that 15%-18% of past participants report a history of violence and even more report a history of non-violent offenses. As part of the LTF study we will have the ability to investigate early predictors of adverse outcomes including suicide, early death, violence, and incarceration. These adverse events, along with the diagnostic outcome heterogeneity further emphasize the vulnerability of individuals who meet CHR criteria and the importance of early identification and prevention of a host of serious outcomes in addition to psychosis.

The LTF sample provides interesting descriptive information about the outcome of individuals who at one time met the CHR criteria. When paired with the extensive longitudinal data collected at baseline across studies, it will be possible to investigate predictors of outcome based on information collected 5-25 years before the LTF assessment. A great deal is already known about short-term predictors of psychotic conversion but now it will be possible to perhaps specify predictors of longer-term conversion, affective vs non-affective psychosis, treatment resistance, functional outcome, violence, and early death. The biomarker data collected in baseline studies can be used in not only prediction analyses but also as a means of understanding the neurobiological underpinnings of different diagnostic and behavioral outcomes. The extensive treatment and substance use information collected at LTF can be used to better understand how these variables might alter the course of illness either positively or negatively. With a better understanding of what predicts resilient outcomes, it will be possible to more specifically focus treatment to bolster those factors associated with better outcomes and more aggressively target domains that may lead to treatment resistance.^50^

There are a few limitations to make note of regarding the current study. First, although we will be able to determine whether there was a follow-up bias in some areas (eg, past converters vs non-converters agreeing to participate) by comparing the LTF database to that of the original studies, it may be impossible to know if there was a bias in terms of other long term outcomes (more people with current affective psychosis vs non-affective psychosis agreeing to participate). It is also not possible to determine at this time whether our preliminary findings are generalizable beyond the NAPLS sites. Future studies that examine LTF will be helpful in this regard.

The LTF data from the collective NAPLS sites will be made public in order for future investigators to explore additional questions to better understand the long term outcome of individuals who meet the CHR criteria and those factors that influence early trajectories of mental illness.

## Supplementary Material

Tables_sbaf133
