# Supplementary material for: Predictors and Moderators of Long-Term Outcome of Persons at Clinical High Risk for Psychosis: Methods and Preliminary Data
Source: Schizophr Bull. 2025 Aug 26;52(4):sbaf133. doi: 10.1093/schbul/sbaf133 (PMC13391608; doi:10.1093/schbul/sbaf133)
Supplement: Tables_sbaf133 [file tables_sbaf133.docx]

| **Supplemental Table 1. Number of CHR Subjects recruited across sites:** | | | | | | | |
| --- | --- | --- | --- | --- | --- | --- | --- |
|  | NAPLS 1 | NAPLS 2 | NAPLS 3 | OMEGA 3 | REGROUP | OTHER | **Total** |
| UCLA | 46 | 108 | 78 | 0 | 0 | 29 | **261** |
| Emory | 9 | 89 | 79 | 10 | 0 | 0 | **187** |
| Harvard | 0 | 56 | 62 | 9 | 0 | 0 | **127** |
| Hillside | 44 | 85 | 78 | 8 | 9 | 20 | **244** |
| UNC | 51 | 91 | 84 | 8 | 0 | 36 | **270** |
| UCSD | 59 | 91 | 65 | 8 | 3 | 3 | **229** |
| Calgary | 0 | 150 | 97 | 1 | 17 | 0 | **265** |
| Yale | 124 | 94 | 85 | 5 | 0 | 77 | **385** |
| UCSF | 134 | 0 | 82 | 0 | 0 | 0 | **216** |
| **Total** | **467** | **764** | **710** | **49** | **29** | **136** | **2184** |

| **Supplemental Table 2. CHR Biomarker Data Available:** | | | | | | |
| --- | --- | --- | --- | --- | --- | --- |
|  | **Electrophysiology** | **Neuroimaging** | **Neurocog** | **Cortisol** | **Inflammatory** | **DNA** |
| NAPLS1 | 89 Startle | 70 Structural MRI | 304 | 9 | 11 | 291 |
| NAPLS2 | 552 P300  579 MMN  499 Startle | 378 Structural MRI  172 fMRI/resting state | 689 | 528 | 62 | 588 |
| NAPLS3 | 516 P300  614 MMN  590 Startle | 573 Structural MRI  571 fMRI/resting state  569 DTI | 620 | 627 | 590 | 620 |

| **Supplemental Table 3. Participants who have already consented to be recontacted for future studies** | | | | | | | | | | |
| --- | --- | --- | --- | --- | --- | --- | --- | --- | --- | --- |
| Site | UCSD | UCLA | UCSF | Calg | Emory | UNC | Harv | Yale | Hillside | Total |
| # of past CHR participants | 229 | 261 | 216 | 265 | 187 | 270 | 127 | 385 | 244 | **2184** |
| # agreed to be recontacted | 133 | 260 | 189 | 0 | 187 | 255 | 103 | 254 | 229 | **1279** |
